# Evaluation of a new point‐of‐care quantitative reverse transcription polymerase chain test for detecting severe acute respiratory syndrome coronavirus 2

**DOI:** 10.1002/jcla.23992

**Published:** 2021-09-14

**Authors:** Yoshiyuki Watanabe, Ritsuko Oikawa, Toshio Suzuki, Hidemitsu Funabashi, Daisuke Asai, Yutaka Hatori, Hiromu Takemura, Hiroyuki Yamamoto, Fumio Itoh

**Affiliations:** ^1^ Department of Internal Medicine Kawasaki Rinko General Hospital Kawasaki Japan; ^2^ Division of Gastroenterology and Hepatology Department of Internal Medicine St. Marianna University School of Medicine Kawasaki Japan; ^3^ Department of Medical Oncology Faculty of Medicine University of Tsukuba Tsukuba Japan; ^4^ Division of Respiratory Medicine Department of Internal Medicine Matsudo City General Hospital Matsudo Japan; ^5^ Department of Microbiology St Marianna University School of Medicine Kawasaki Japan; ^6^ Department of Internal Medicine Hatori Clinic Kawasaki Japan

**Keywords:** COVID‐19, POCT, qRT‐PCR, quenching probe, SARS‐CoV‐2

## Abstract

**Background:**

Severe acute respiratory syndrome coronavirus 2 (SARS‐CoV‐2) infection is rapidly spreading worldwide, and the resultant disease, coronavirus disease (COVID‐19), has become a global pandemic. Although there are multiple methods for detecting SARS‐CoV‐2, there are some issues with such tests, including long processing time, expense, low sensitivity, complexity, risk of contamination, and user friendly. This study evaluated the reproducibility and usability of a new point‐of‐care test (POCT) using real‐time quantitative reverse transcription polymerase chain reaction (qRT‐PCR) for detecting SARS‐CoV‐2.

**Methods:**

Samples from 96 patients with suspected SARS‐CoV‐2 infection were assessed using the real‐time qRT‐PCR‐based POCT and the conventional real‐time qRT‐PCR method based on the Japanese National Institute of Infectious Diseases guidelines (registration number: jRCT1032200025).

**Results:**

The real‐time qRT‐PCR‐based POCT had a positive agreement rate of 90.0% (18/20), a negative agreement rate of 100% (76/76), and a total agreement rate of 97.9% (94/96), and the significantly high score of questionnaire survey (total score *p* < 0.0001). In the two cases in which real‐time qRT‐PCR‐based POCT results did not match conventional real‐time qRT‐PCR test results, the SARS‐CoV‐2 RNA copy numbers were 8.0 copies per test in one case and below the detection limit in the other case when quantified using conventional real‐time qRT‐PCR. All patients could be triaged within 1 day using the real‐time qRT‐PCR‐based POCT without invalid reports.

**Conclusions:**

The real‐time qRT‐PCR‐based POCT not only had high reproducibility and useability but also allowed rapid patient triage. Therefore, it may be helpful in clinical settings.

## Graphical abstract

We assessed a new point‐of‐care test that uses the quantitative reverse transcription polymerase chain reaction (qRT‐PCR). This test reduces the extra RNA purification step, allowing much faster results. We compared the results of this test to those of the traditional qRT‐PCR test.

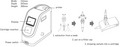

## 1 INTRODUCTION

Coronavirus disease (COVID‐19) is an acute respiratory infection caused by the novel severe acute respiratory syndrome coronavirus 2 (SARS‐CoV‐2).^1^ It was first reported as “pneumonia of unknown cause” in Wuhan, China, in December 2019, and the Chinese Centers for Disease Control and Prevention (CDC) officially announced on January 7, 2020, that COVID‐19 was caused by the novel coronavirus.

There are some molecular diagnostic tools for detecting SARS‐CoV‐2, such as the standard quantitative reverse transcription polymerase chain reaction (qRT‐PCR) test issued by the CDC in the United States,^2^ the standard qRT‐PCR test issued by the National Institute of Infectious Diseases (NIID) in Japan,^3^ the high‐throughput qRT‐PCR kit test, the reverse transcription loop‐mediated isothermal amplification test,^4^ the viral antigen test,^5^ the quantitative viral antigen test, and the viral antibody test.^6^, ^7^ There are also multiple sample collection methods, such as collection using nasopharyngeal swabs or saliva.^8^ As all of these methods have both strengths and limitations, it is necessary to choose among them based on clinical requirements. Although the standard method is a qRT‐PCR test from a nasopharyngeal swab, it has some limitations in clinical use, because of including long processing time, expense, low positive agreement rate, complexity, user friendly, and a level of personal protective equipment (PPE).

In this study, a point‐of‐care test (POCT) using a real‐time one‐step qRT‐PCR method based on the existing Japanese test method “Manual for the Detection of Pathogen 2019‐nCoV” was performed using nasopharyngeal swab specimens collected from patients with suspected COVID‐19.^3^ We compared our results with those of the existing method to assess its performance. In addition, we assessed the correlation between the RNA copy number using conventional real‐time qRT‐PCR and the cycle threshold in the real‐time qRT‐PCR‐based POCT to determine the positive agreement rate of the evaluation kit in detecting small amounts of SARS‐CoV‐2.

## 2 MATERIAL AND METHODS

### 2.1 Patients

Clinical study was performed at the Kawasaki Rinko General Hospital and the Matsudo City General Hospital during a 3‐month period (April 30, 2020, to July 20, 2020). The study was prospective observation study, carried out by the opt‐in method of each institution, and approved by the ethics committees of the Japan Medical Association (approval code: #R2‐03) and Matsudo City General Hospital (approval code: #0014), and informed consent for specimen collection and testing was obtained from all participants. Details of our study design and results are uploaded to the Japan Registry of Clinical Trials (registration number: jRCT1032200025, scientific title: Clinical evaluation of the SARS‐CoV‐2 detection system (COVID‐19), type of the clinical trial: observational study) website (https://jrct.niph.go.jp/re/reports/detail/7882).

Samples were collected from 96 patients suspected to have COVID‐19 based on fever, cough, pneumonia, or imaging diagnosis; who had a history of close contact with an infected person; or who were cured from SARS‐CoV‐2 infection. Two nasopharyngeal swab specimens were collected from each patient: one was suspended in 1 ml of liquid culture media (UTM), and the other was suspended in the extraction reagent solution for the evaluation kit.^9^ Questionnaire survey was conducted for all 6 laboratory staffs in charge involved in the analysis. There are 5 kinds of questions (1. Operability, 2. Contribution to reducing infection risk, 3. Ease of result judgment, 4. Error handling, and 5. Total satisfaction level) and was 5 grades for each question.

### 2.2 Conventional real‐time qRT‐PCR

RNA purification and real‐time one‐step qRT‐PCR (conventional real‐time qRT‐PCR) were performed for specimens collected in UTM using a method based on the “Manual for the Detection of Pathogen 2019‐nCoV” Ver.2.9.1 issued by the NIID.^3^

### 2.3 Real‐time qRT‐PCR‐based POCT test using new evaluation kit for rapid gene detection

This evaluation kit allows the user to perform RNA purification, reverse transcription, amplification, and detection of SARS‐CoV‐2 RNA (real‐time qRT‐PCR‐based POCT). The kit includes a test cartridge, extraction reagent solution, filters, and collection swabs and is used with a dedicated device. The operation method is described below (Mizuho Medy Co., Ltd.).

**FIGURE 1:**
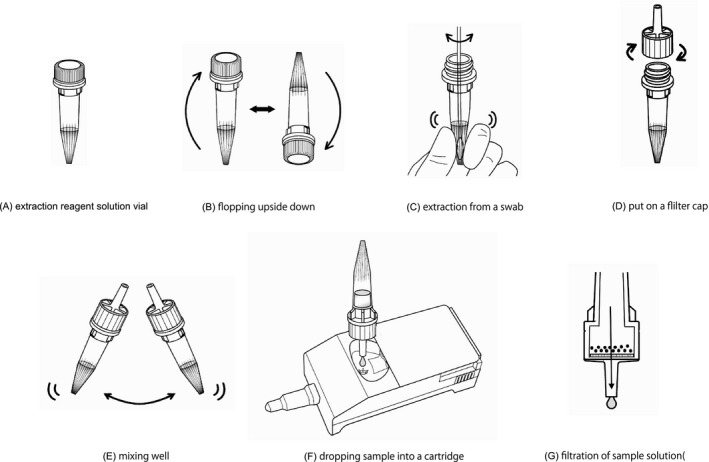
Operation method of evaluation reagent (real‐time qRT‐PCR‐based POCT). The operation is performed according to (A) to (G) in the figure

The extraction reagent solution (Figure 1A) is composed of detergents, chaotropic salts (guanidium ions), and silica particles. The particles are dispersed before insertion of the swab by inverting the vial five times to mix the extraction reagent solution (Figure 1B). RNA extraction is performed by inserting the tip of the swab on which the sample was collected into the bottom of the extraction reagent solution vial, holding the tip from the outside of the vial so that the surface of the tip lightly touches the inside of the vial, and rotating the tip left and right about five times each (Figure 1C). Then, the filter is tightened (Figure 1D), and the vial is shaken several times to thoroughly mix the sample (Figure 1E). Four drops of this sample are placed onto the sample spot of the test cartridge (Figure 1F). After the sample drop is absorbed, the test cartridge is promptly inserted into the device, which begins the measurement.

In general, a complicated purification step is required when amplifying RNA because impurities present in the sample, such as human genomic DNA, inhibit the reaction. In this method, DNA binds to silica particles in the extraction reagent solution, and these particles are removed by filtration. Thus, the target RNA is extracted into the droplet solution and easily measured (Figure 1G).

SARS‐CoV‐2 RNA in the dropped sample binds to silica particles on a membrane in the mouth of the test cartridge's sample spot and is washed and transferred to the reaction tube for reverse transcription, followed by PCR and fluorescence detection. All operations after dropping the sample are performed within approximately 1 h in the dedicated fully automated gene analysis device Smart Gene (Mizuho Medy Co., Ltd.).

**FIGURE 2:**
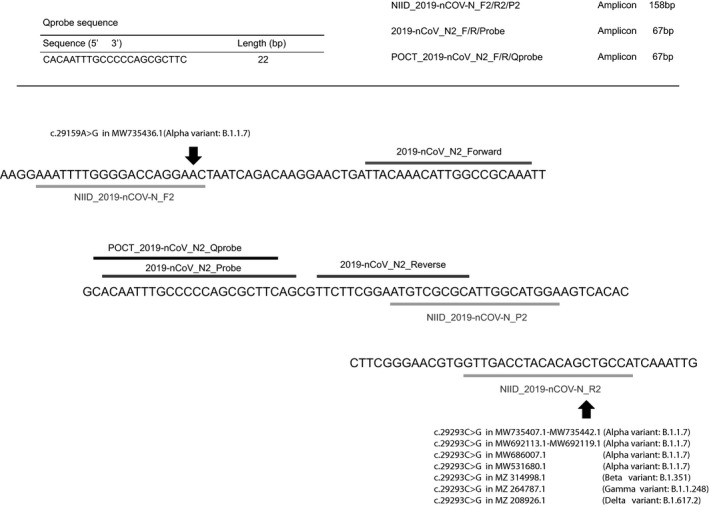
Target sequence of real‐time qRT‐PCR for SARS‐CoV‐2 detection. There are two kinds of primers and probe set. NIID‐based primers show light gray bar (NIID‐2019‐nCOV‐N_F2, NIID‐2019‐nCOV‐N_R2 and NIID‐2019‐nCOV‐N_P2). CDC‐based primers show gray bar (2019‐nCoV_N2_Forward, 2019‐nCoV_N2_Reverse, 2019‐nCoV_N2_Probe). In real‐time qRT‐PCR‐based POCT method, Qprobe shows darker gray bar and primers are same as CDC‐based primer. Add single nucleotide polymorphism in the binding site of these CDC‐based primers and NIID‐based primers in SARS‐CoV‐2 mutant strains

In this test, the target gene is the SARS‐CoV‐2 gene nucleocapsid protein N (N‐Nucleocapsid phosphoprotein). Reverse transcription and amplification by real‐time one‐step RT‐PCR using N2 primers and detection by a quenching probe (QProbe, Mizuho Medy Co., Ltd.) are performed to measure the number of copies of SARS‐CoV‐2 RNA, as stated in “2019‐Novel Coronavirus (2019‐) nCoV) Real‐time RT‐PCR Panel Primers and Probes” (Effective: 24 Jan 2020), published by the US CDC.^2^ Reverse transcription is performed at 54°C for 10 min, followed by 45 cycles of amplification of 10 s at 100°C and 20 s at 56°C. The QProbe binds to the amplified target sequence at a specific temperature and quenches the labeled fluorescent material. If quenching of the amplified product is observed, the positive result and number of cycles determined to be positive are printed out and displayed on the monitor. Figure 2 shows the sequence used for QProbe, and the 3′ end of the probe is labeled with the fluorescent dye BODIPY FL (Mizuho Medy Co., Ltd.).

### 2.4 Measurement of SARS‐CoV‐2 RNA copy number

SARS‐CoV‐2 RNA was extracted from the residual sample of the evaluation kit using the QIAamp DNA Ⅿini Kit (Qiagen; Hilden), as in the control method, and the extracted RNA was used to quantitatively measure the SARS‐CoV‐2 RNA copy number from the calibration curve of N2 primers as previously described.^3^

### 2.5 Statistical analysis

**FIGURE 3:**
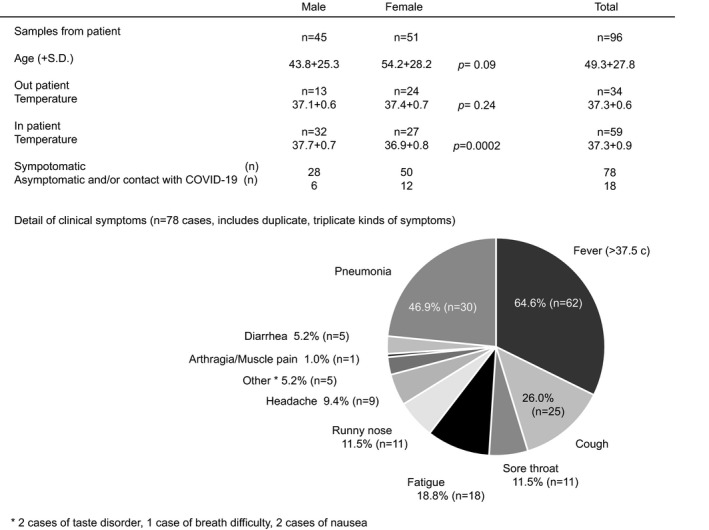
Characteristics and clinical symptoms of the subjects. Clinical characteristics shows 5 factors (sample number, age, body temperature in outpatient, body temperature in inpatient, and symptomatic or asymptomatic). The detail of the symptoms is shown as pie chart

**FIGURE 4:**
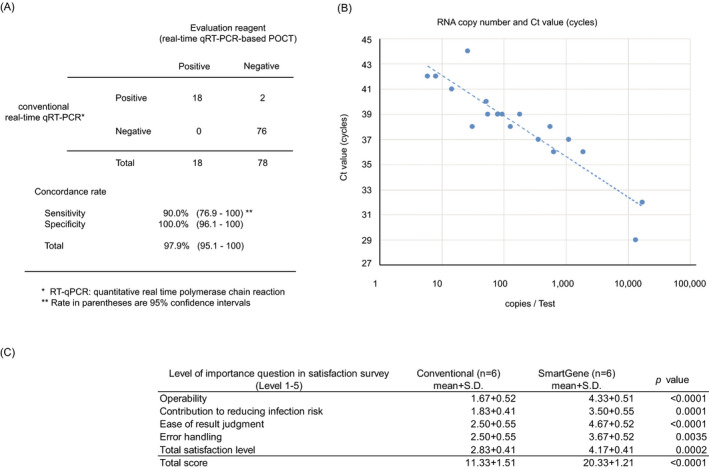
Positive/Negative agreement rate of the Target sequence of real‐time qRT‐PCR for SARS‐CoV‐2 detection. (A) Sensitivity and specificity of the evaluation reagent compared to RT‐qPCR results with COVID‐19. (B) Correlation between conventional real‐time qRT‐PCR and real‐time qRT‐PCR‐based POCT in 18 SARS‐CoV‐2 positive cases. RNA copy number and Ct value of evaluation reagent (cycles). (C) Results of questionnaire survey of laboratory staffs (n = 6)

Clinical characteristics and symptoms were counted and calculated statistical analysis each male and female using the unpaired t test (Figure 3). Questionnaire survey were a calculated statistical analysis using the unpaired t test (Figure 4C). Forward, reverse, and Qprobe sequencing data were analyzed and visualized using Geneious Prime software (v.2019.2.3; (Biomatters Ltd) (Figure 2). Positive agreement rate and negative agreement rate of the real‐time qRT‐PCR‐based POCT result compared to conventional real‐time qRT‐PCR result were calculated statistical analysis using the unpaired Fisher's exact test (Figure 4A). All statistical analyses were performed using SPSS for Windows (v.12.0; SPSS, Inc.) and PRISM for Windows (v.7.0; GraphPad Software). No adjustment of multiple comparisons was made. All reported *p*‐values were two‐sided, and a *p* < 0.05 was considered significant.

## 3 RESULTS

### 3.1 Clinical samples

In total, 96 patients who had a history of close contact with an infected person; experienced fever, cough, or pneumonia; or were cured from infection were included in our study. There were no significant differences in age or body temperature of individuals examined as outpatients based on gender; however, the body temperatures of male patients who were hospitalized were significantly higher than female patients (*p* = 0.0002). Patient details, including age, sex, patient background, and clinical symptoms, are shown in Figure 3. Seventy‐eight patients had at least one symptom, with many having two or three symptoms. The most common symptoms were fever (n = 62, 64.6%) and pneumonia (n = 30, 46.9%) (Figure 3). Details of results data are linked to the Japan Registry of Clinical Trials website (registration number: jRCT1032200025, scientific title: Clinical evaluation of the SARS‐CoV‐2 detection system (COVID‐19), type of the clinical trial: observational study) and are available to share and download the files to all person who is interested (https://jrct.niph.go.jp/re/reports/detail/7882), includes pdf format raw data, study protocol, statistical analysis plan, informed consent form, and clinical study report (from August 1, 2021).

### 3.2 Correlation of results between the conventional and the evaluation of real‐time qRT‐PCR test

We compared the results of the conventional real‐time qRT‐PCR method with real‐time qRT‐PCR‐based POCT method using 96 nasopharyngeal swab suspensions. Using the conventional real‐time qRT‐PCR method, 20 cases were positive and 76 were negative. Using the real‐time qRT‐PCR‐based POCT method, 18 cases were positive and 78 were negative. Therefore, based on the results of the conventional real‐time qRT‐PCR method, the positive agreement rate of the real‐time qRT‐PCR‐based POCT method was 90.0% (18/20) (Figure 4A) and the negative agreement rate was 100.0% (76/76) (Figure 4A).

The relationship between the number of SARS‐CoV‐2 RNA copies and the cycle number when quenching for the 18 positive samples is summarized in Figure 4A. There was a strong correlation (*r* = 0.91), and the limit of detection was estimated to be 5–10 copies per test (Figure 4B). In the two cases in which real‐time qRT‐PCR‐based POCT results did not match conventional real‐time qRT‐PCR test results, the SARS‐CoV‐2 RNA copy numbers were 8.0 copies per test in one case and below the detection limit in the other case when quantified using conventional real‐time qRT‐PCR.

Moreover, all patients successfully received medical triage from clinical doctors because they could undergo a test on the same day using the real‐time qRT‐PCR‐based POCT (minimum, less than 1 h).

### 3.3 Validation of the nucleotide variance of primer binding sites in SARS‐CoV‐2 mutant strains

To validate nucleotide variance of these CDC‐based and NIID‐based primer binding sites in already reported SARS‐CoV‐2 mutant strains, we found some single nucleotide polymorphism (alpha [B.1.1.7], c.29159A > G in MW735436.1, c.29293C > G in MW735407.1–735442.1, MW692113.1–692119.1, MW686007.1, MW531680.1: beta [B.1.351], c.29293C > G in MZ314998.1: gamma [B.1.1.248] c.29293C > G in MZ264787.1 and delta [B.1.617.2] c.29293C > G in MZ208926.1) in NIID‐based primer binding site (Figure 2).

### 3.4 Level of importance question in your satisfaction survey

Questionnaire survey was conducted for all 6 laboratory staffs in charge involved in the analysis. Real‐time qRT‐PCR‐based POCT is not only significantly higher than conventional real‐time qRT‐PCR test in total score, but also every 5 questions (Figure 4C).

## 4 DISCUSSION

We successfully evaluated the reproducibility and usability of a new point‐of‐care test (POCT) using real‐time qRT‐PCR for detecting SARS‐CoV‐2 (Real‐time qRT‐PCR‐based POCT). Our real‐time qRT‐PCR‐based POCT method was found to be as high accurate (high sensitivity, specificity, and reproducibility) as conventional real‐time qRT‐PCR without invalid reports in these cases. Moreover, we found real‐time qRT‐PCR‐based POCT has the potential of high usability as a POCT by questionnaire survey. The usefulness of this device in clinical use (bedside) is to be high.

In this study, a point‐of‐care test (POCT) using a real‐time one‐step qRT‐PCR method based on the existing Japanese test method “Manual for the Detection of Pathogen 2019‐nCoV” was performed using nasopharyngeal swab specimens collected from patients with suspected COVID‐19.^3^ We compared our results with those of the existing method to assess its performance. In addition, we assessed the correlation between the RNA copy number using conventional real‐time qRT‐PCR and the cycle threshold in the real‐time qRT‐PCR‐based POCT to determine the positive agreement rate of the evaluation kit in detecting small amounts of SARS‐CoV‐2.

According to the recommendations of the Japanese Association for Infectious Diseases and the Japanese Society for Environmental Infectious Diseases, gene detection methods such as real‐time qRT‐PCR using a nasopharyngeal swab, saliva, or sputum samples are the preferred testing methods for SARS‐CoV‐2. In addition, the use of antibody detection by immunochromatography in the blood or serum is being evaluated. Similarly, an antigen detection method based on immunochromatography using a nasopharyngeal swab solution has been approved by the Ministry of Health, Labour and Welfare in Japan. These testing systems are being expanded to take advantage of the characteristics of each method. However, these testing systems also have inadequacies that limit their broad use. Immunochromatographic antibody testing requires an increase in antibodies approximately 2 weeks after the onset of symptoms, and non‐specific reactions are also observed, which poses a challenge for diagnosis in the acute phase of the disease and requires careful consideration of the overall clinical condition. The antigen test requires a considerably higher viral load for detection than the real‐time qRT‐PCR test, and it is not considered to have adequate detection performance for screening asymptomatic individuals or confirming a negative result in the convalescence period when only a small amount of virus is shed.^10^ SARS‐CoV‐2 detection using real‐time qRT‐PCR has been covered by government‐based medical insurance since March 2020; however, currently, it is only available at medical institutions and laboratories that offer outpatient services for returnees and close contacts after consultation with the Returnee and Contact Consultation Center in each prefecture. Although there is an urgent need to expand the PCR testing system,^11^ even if general medical institutions are allowed to perform real‐time qRT‐PCR testing, general practitioners will have to outsource testing because of the need for expensive and specialized equipment and expertise in testing techniques such as specimen extraction.

Some other PCR‐based POCT method for SARS‐CoV‐2 detection was seen recently, such as LAMP (Loop‐mediated isothermal amplification) and NEAR (Nicking Endonuclease Amplification Reaction) isothermal amplification method.^12^, ^13^ These methods have advantages such as not only rapid detection (around 30 min for the whole process) but also high specificity using multiple primers (four or more primers) than the standard PCR method. However, SARS‐CoV‐2 (RNA virus) frequently changes any nucleotides not only spike (S) protein but also nucleocapsid (N) protein region. Interestingly, SARS‐CoV‐2 mutant strains (alpha, beta, gamma, and delta strains) already show some nucleotide variance in NIID‐based sense/ antisense primer binding site referenced from NCBI (National Center for Biotechnology Information) (Figure 2), suggesting it may affect efficient gene amplification with multiple primers in mutant strains. Moreover, invalid reports were seen caused by sample quality, amplification inhibitors, extraction issues, and identification algorism of the appropriate amplification),^14^ especially the judgment of positive or negative is the most important factor for the high usability POCT.

Therefore, real‐time qRT‐PCR equipment that can be used at the bedside, not the bench side, a so‐called POCT, is desperately needed. Recently, the “reagent for rapid gene detection of SARS‐CoV‐2 as a POCT” (Mizuho Medy Co., Ltd.), a dedicated kit for the fully automated gene analysis device Smart Gene, was developed. This kit only requires one‐step specimen preparation (no need to prepare and add any reactive regents). The swab containing the nasopharyngeal specimen is suspended in the extraction reagent, and the reagent is then dropped into the test cartridge (prefilled all PCR mixture reagent, primers, Q‐probes, reverse transcriptase); therefore, there is little contamination or risk of infection. SARS‐CoV‐2 RNA can then be detected by simply placing the test cartridge in the device (Figure 1A‐G). This qPCR machine (includes kit) is also cost effective (approximately $4300) and short measurement time (less than 60 min). Therefore, a diagnosis can be made at the point of care without making patients wait. The evaluation kit can also be used to diagnose infection in patients, provide an appropriate indicator for the prevention of nosocomial infections, and protect medical personnel on the front lines of medicine. Therefore, allowing its practical use is also extremely important in clinical medicine. However, we not yet have an enough evidence because of limitation of our study size.

## 5 CONCLUSION

We compared the conventional real‐time qRT‐PCR and a real‐time qRT‐PCR‐based POCT for the detection of SARS‐CoV‐2. The real‐time qRT‐PCR‐based POCT showed high positive agreement rate (90.0%) and negative agreement rate (100.0%), as well as a sensitive correlation between RNA copy number and Ct values (*r* = 0.91), when compared with the conventional method. Moreover, all patients successfully received medical triage from clinical doctors because they could undergo a test on the same day using the real‐time qRT‐PCR‐based POCT (minimum, less than 1 h). Therefore, we believe this new POCT using real‐time qRT‐PCR is the best method for clinical use.

## CONFLICT OF INTEREST

The authors state that they have no conflict of interest.

## AUTHOR CONTRIBUTIONS

Watanabe Y and Funabashi H designed and coordinated the study and performed the experiments, acquired, and analyzed data; Watanabe Y, Oikawa R, Suzuki T, Funabashi H, Asai D, Hatori Y, Takemura H, and Yamamoto H interpreted the data; Watanabe Y, Oikawa R, Funabashi; Watanabe Y validated mutant strains using bioinformatics approach; H, Yamamoto H, Itoh F wrote or edited the article; all authors approved the final version of the article.

## APPROVAL CODE ISSUED BY THE INSTITUTIONAL REVIEW BOARD (IRB)

The study was prospective observation study, carried out by the opt‐in method of each institution, and approved by the ethics committees of the Japan Medical Association (approval code: #R2‐03) and Matsudo City General Hospital (approval code: #0014), and informed consent for specimen collection and testing was obtained from all participants. Details of our study design and results are uploaded to the Japan Registry of Clinical Trials website (registration number: jRCT1032200025, scientific title: Clinical evaluation of the SARS‐CoV‐2 detection system (COVID‐19), type of the clinical trial: observational study) (https://jrct.niph.go.jp/re/reports/detail/7882) and are available to share and download the files to all person who is interested (https://jrct.niph.go.jp/re/reports/detail/7882), includes pdf format raw data, study protocol, statistical analysis plan, informed consent form, and clinical study report (from August 1 2021).

## Data Availability

The data that provided the evidence for the study are available from the corresponding author upon reasonable request.
